# Comparative Transcriptome Analysis of Rutabaga (*Brassica napus*) Cultivars Indicates Activation of Salicylic Acid and Ethylene-Mediated Defenses in Response to *Plasmodiophora brassicae*

**DOI:** 10.3390/ijms21218381

**Published:** 2020-11-08

**Authors:** Qinqin Zhou, Leonardo Galindo-González, Victor Manolii, Sheau-Fang Hwang, Stephen E. Strelkov

**Affiliations:** Department of Agricultural, Food and Nutritional Science, University of Alberta, Edmonton, AB T6G 2P5, Canada; qinqin@ualberta.ca (Q.Z.); galindo@ualberta.ca (L.G.-G.); vmanolii@ualberta.ca (V.M.); sheau.fang.hwang@ualberta.ca (S.-F.H.)

**Keywords:** *Brassica napus*, *Plasmodiophora brassicae*, clubroot, RNA sequencing (RNA-seq), defense mechanisms, salicylic acid, ethylene

## Abstract

Clubroot, caused by *Plasmodiophora brassicae* Woronin, is an important soilborne disease of *Brassica napus* L. and other crucifers. To improve understanding of the mechanisms of resistance and pathogenesis in the clubroot pathosystem, the rutabaga (*B. napus* subsp. *rapifera* Metzg) cultivars ‘Wilhelmsburger’ (resistant) and ‘Laurentian’ (susceptible) were inoculated with *P. brassicae* pathotype 3A and their transcriptomes were analyzed at 7, 14, and 21 days after inoculation (dai) by RNA sequencing (RNA-seq). Thousands of transcripts with significant changes in expression were identified in each host at each time-point in inoculated vs. non-inoculated plants. Molecular responses at 7 and 14 dai supported clear differences in the clubroot response mechanisms of the two genotypes. Both the resistant and the susceptible cultivars activated receptor-like protein (*RLP*) genes, resistance (*R*) genes, and genes involved in salicylic acid (SA) signaling as clubroot defense mechanisms. In addition, genes related to calcium signaling and genes encoding leucine-rich repeat (LRR) receptor kinases, the respiratory burst oxidase homolog (RBOH) protein, and transcription factors such as WRKYs, ethylene responsive factors, and basic leucine zippers (bZIPs), appeared to be upregulated in ‘Wilhelmsburger’ to restrict *P. brassicae* development. Some of these genes are essential components of molecular defenses, including ethylene (ET) signaling and the oxidative burst. Our study highlights the importance of activation of genes associated with SA- and ET-mediated responses in the resistant cultivar. A set of candidate genes showing contrasting patterns of expression between the resistant and susceptible cultivars was identified and includes potential targets for further study and validation through approaches such as gene editing.

## 1. Introduction

Clubroot, caused by the obligate parasite *Plasmodiophora brassicae* Woronin, is an important soilborne disease of Brassica crops. Susceptible plants develop characteristic root galls following infection, which interrupt water and nutrient uptake and result in significant yield and quality losses. Globally, losses from clubroot have been estimated at 10–15% [1]. In Canada, the disease has long been an issue on cruciferous vegetables [2], and since the early 2000s has emerged as an important constraint to the production of canola (oilseed rape; *Brassica napus* L.) [3]. As canola is one of the most valuable crops for Canadian farmers, contributing $26.7 billion CAD annually to the national economy [4], there have been significant efforts to improve the understanding and management of this disease. While numerous control strategies have been evaluated, including long rotations out of susceptible hosts and the application of soil amendments to reduce disease pressure [5,6], the deployment of genetically resistant canola cultivars remains the backbone of clubroot management [7].

The first clubroot resistant (CR) canola cultivars were introduced to Canada in 2009–2010, and at present there are about 30 CR varieties from various seed companies on the market [8]. The basis of this resistance, however, appears to be similar across most cultivars, and is derived from the European winter *B. napus* ‘Mendel’ [9]. Although ‘Mendel’-type resistance initially provided excellent protection against all pathotypes of *P. brassicae* known in Canada, it was first overcome in 2013 [10], just four years after its introduction. Subsequent studies have documented the loss or erosion of resistance in an increasing number of fields, likely because of selection pressure imposed by CR canola on *P. brassicae* populations [11,12]. This has resulted in the emergence of multiple ‘novel’ pathotypes of *P. brassicae* that are highly virulent on CR canola; among these, pathotype 3A, as defined on the Canadian Clubroot Differential (CCD) set, is predominant in western Canada, where most canola is grown [11]. New sources of clubroot resistance, combined with other management strategies and better understanding of resistance mechanisms, will improve long-term control of this disease.

Plants have a two-layer immune system for defense against pathogen attack. Pathogen-associated molecular pattern (PAMP)-triggered immunity (PTI) is the first line of defense to generic pathogen signals [13]. This first line of defense is initiated by pattern recognition receptors (PRRs), usually receptor kinases and receptor-like proteins (RLPs), which recognize evolutionarily conserved PAMPs or endogenous damage-associated molecular patterns (DAMPs) [14]. Pathogens can, however, suppress PTI and facilitate virulence via the production of specific effectors. These effectors can be detected by specific resistance (*R*) genes in the plant in a “gene-for-gene” type interaction, activating the second layer of immunity, called effector-triggered immunity (ETI) [13]. The *R*-gene response has been studied more extensively in the clubroot pathosystem and has proven important for resistance to this disease. For example, two clubroot resistance genes, *CRa* and *Crr1*, cloned in *B. rapa*, encode Toll-interleukin receptor nucleotide-binding site leucine-rich repeat (TIR-NBS-LRR) proteins, characterized as *R* genes [15,16]. In each of *B. napus*, *B. oleracea*, and *B. rapa*, around 10–20 quantitative resistance loci (QTL) have been mapped for clubroot resistance [17]. Various additional *R* genes have been identified in clubroot resistance loci in *B. rapa*, including *Crd*, *Rcr1*, *Rcr2*, *Rcr4*, *Rcr6*, *Rcr8*, and *Rcr9* [18,19,20,21,22], which could be important resources for resistance breeding. Recently, there has been increasing interest in identifying and utilizing PTI-related genes in QTL to achieve long-term resistance to many diseases [23]. Therefore, key resistance regulators beyond *R* genes also have the potential for use in clubroot resistance breeding programs.

Transcriptomic analyses have been conducted with increasing frequency in the study of *P. brassicae*-host interactions. For instance, recent transcriptomic studies of the responses of *B. rapa* and *B. juncea* to the clubroot pathogen have suggested the involvement of PTI and ETI in resistant reactions. These responses included the activation of genes encoding PRRs, R proteins, mitogen-activated protein kinases (MAPK), transcription factors (TFs), pathogenesis-related (PR) proteins, as well as genes involved in cell wall modification, calcium, and hormone signaling, and the production of reactive oxygen species (ROS) [24,25]. In another study comparing the transcriptomes of clubroot susceptible (CS) and CR *B. napus* lines carrying resistance introgressed from rutabaga (*B. napus* subsp. *rapifera* Metzg), long noncoding RNAs appeared to be involved in regulating target genes involved in the plant-pathogen interaction, hormone signaling, and primary/secondary metabolism in response to *P. brassicae* [26]. Studies with rutabaga are particularly relevant for understanding the interaction between the clubroot pathogen and canola, since rutabaga is a source of resistance for the latter [27,28,29,30].

A recent study investigating the transcriptomes of *B. napus* cultivars with differential resistance to *P. brassicae* pathotype 5X indicated the involvement of salicylic acid (SA)-mediated immunity in the resistance expressed by the cultivar ‘Laurentian’ [31]. This cultivar, however, is susceptible to pathotype 3A, the predominant resistance-breaking pathotype in western Canada [11]. In the current study, to better understand host responses to *P. brassicae* and identify candidate genes for canola clubroot resistance breeding, we challenged the rutabagas ‘Wilhelmsburger’ and ‘Laurentian’ with pathotype 3A of *P. brassicae* and compared their transcriptomic responses at multiple time-points during secondary infection. Both the resistant (‘Wilhelmsburger’) and susceptible (‘Laurentian’) cultivars activated *RLP* genes, *R* genes, and genes involved in SA synthesis and signaling, in response to the pathogen. The resistant host, however, also appeared to coordinate the activity of genes involved in various additional pathways, including ethylene (ET) signaling. This study provides insights on possible common defense responses mediated by rutabaga cultivars against pathotype 3A, and highlights the molecular defense mechanisms in a specific host (‘Wilhelmsburger’) that could be involved in resistance. Several key genes were identified that may serve as good candidates for future clubroot resistance breeding studies, including functional validation and increased resistance through gene editing.

## 2. Results and Discussion

### 2.1. Disease Assessment

**Figure 1:**
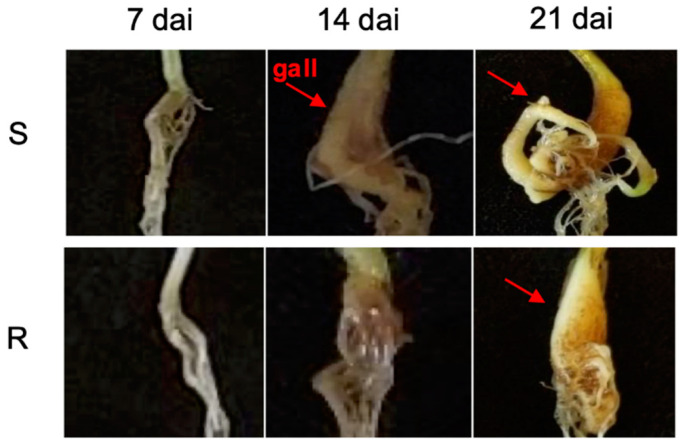
Phenotypes of *Plasmodiophora brassicae*-inoculated roots of the rutabagas ‘Wilhelmsburger’ (R) and ‘Laurentian’ (S) at 7, 14, and 21 days after inoculation (dai). A red arrow indicates the presence of galls.

Clubroot development in both host cultivars following *P. brassicae* inoculation was evaluated based on the severity of root galling. Noticeable galls appeared 14 dai in ‘Laurentian’ but were not visible in ‘Wilhelmsburger’ until 21 dai (Figure 1). At 45 dai, the disease index (DI) on ‘Laurentian’ was 99%, indicating complete susceptibility, while on ‘Wilhelmsburger’ the DI was 48%. This suggested that disease development progressed more slowly, and was not as severe, in ‘Wilhelmsburger.’ These results are consistent with the previously reported reactions of these hosts to *P. brassicae* pathotype 3A [11]. As expected, the susceptible check, Chinese cabbage ‘Granaat,’ developed severe clubroot (DI = 100% at 45 dai).

### 2.2. RNA-Seq Analysis

RNA sequencing (RNA-Seq) was used to assess transcriptional changes between control and inoculated plants at 7, 14, and 21 dai. On average, 38 million reads were generated from 36 cDNA libraries. From these reads, 82.14–90.76% were aligned to the reference genome of *B. napus*. Principal component analysis (PCA) showed consistency among replicates and good separation between inoculated and non-inoculated samples (Supplementary Figure S1).

**Figure 2:**
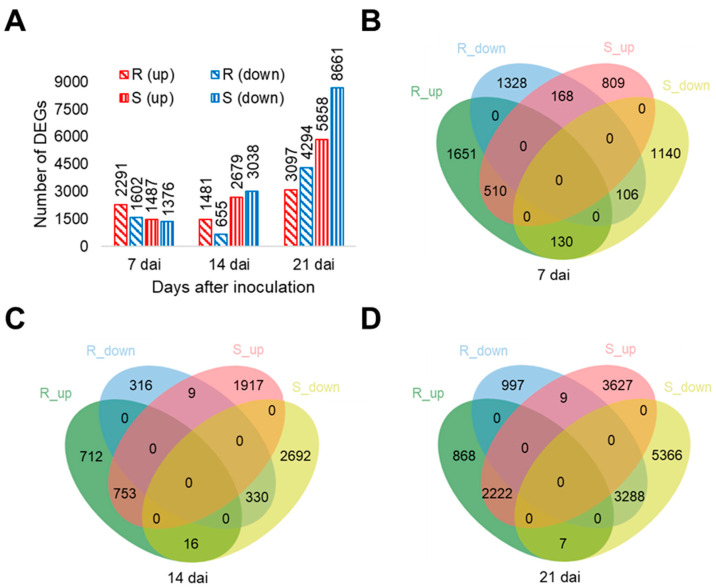
Number of differentially expressed transcripts in each rutabaga cultivar and time-point. (**A**) Number of total differentially expressed transcripts in each cultivar and time-point. (**B–D**) Venn diagrams showing the number of transcripts with common and unique expression patterns in the two cultivars at 7 (**B**), 14 (**C**) and 21 (**D**) days after inoculation (dai). Up, upregulation; down, downregulation; R, the resistant cultivar ‘Wilhelmsburger’; S, the susceptible cultivar ‘Laurentian’.

In total, 110,069 transcripts were identified across samples, which were annotated based on similarity to *B. napus* and Arabidopsis genes. Among these, 20,466 transcripts showed significant expression changes in at least one of six comparison sets of inoculated vs. non-inoculated samples (Supplementary Table S1). Thousands of transcripts were significantly differentially expressed at each time-point. At 7 dai, when no disease symptoms were yet visible in either host, more genes were significantly regulated in ‘Wilhelmsburger’ (3893) than ‘Laurentian’ (2863). At that same time-point, more upregulated genes than downregulated genes were identified in both hosts (Figure 2A). Similarly, when challenging two *B. napus* hosts with *P. brassicae* pathotype 5X, more genes were significantly regulated in the resistant host than in the susceptible host at 7 dai [31]. At 14 dai, although fewer genes were significantly regulated in ‘Wilhelmsburger’ (2136) than ‘Laurentian’ (5717), the patterns of regulation were different in the two hosts. Around two-thirds of the genes were upregulated in the former, but more than half were downregulated in the latter (Figure 2A). At 21 dai, more significantly regulated genes were identified in ‘Laurentian’ (14,519) than ‘Wilhelmsburger’ (7391), with more downregulated genes than upregulated genes in both hosts (Figure 2A). A similar trend was reported by Galindo-González et al. (2020) in susceptible and resistant hosts at 21 dai [31]. In addition, 298, 25, and 16 transcripts showed opposite patterns of regulation in the two hosts at 7, 14, and 21 dai, respectively (Figure 2B–D). These genes could be key regulators of responses associated with clubroot resistance or susceptibility. The potential relevance of some of these genes in the differential host responses is discussed below.

### 2.3. Validation of RNA-Seq Data by Quantitative Real-Time PCR (qRT-PCR)

**Figure 3:**
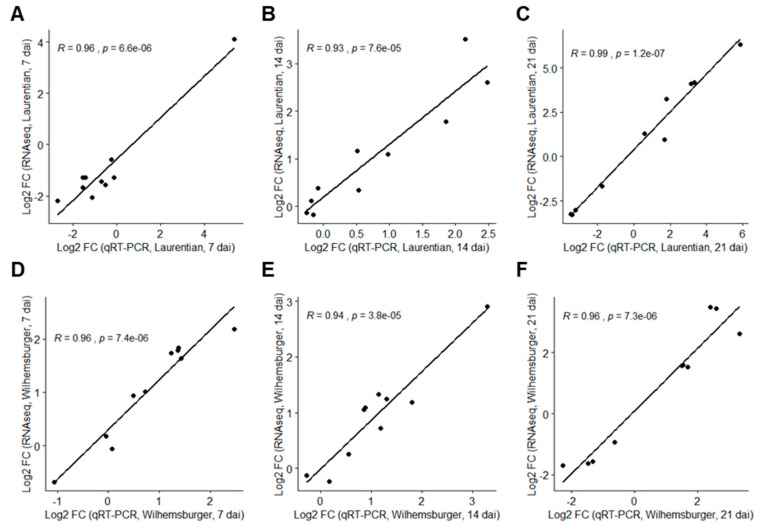
Correlation of log_2_ (fold-change) values of 10 selected genes based on RNA-seq and qRT-PCR analyses (inoculated versus non-inoculated). The R values indicate the correlation coefficient between the two methods in each host and time-point, and the *p*-values indicate the significance level of the *t*-test. (**A**) ‘Laurentian,’ 7 dai; (**B**) ‘Laurentian,’ 14 dai; (**C**) ‘Laurentian,’ 21 dai; (**D**) ‘Wilhelmsburger,’ 7 dai; (**E**) ‘Wilhelmsburger,’ 14 dai; (**F**) ‘Wilhelmsburger,’ 21 dai.

The expression of 10 target genes from each cultivar at each time-point (*P. brassicae* inoculated samples vs. non-inoculated samples) was evaluated by qRT-PCR analysis to validate the RNA-seq results. The resulting absolute log_2_ fold-change (log_2_FC) from RNA-seq and qRT-PCR indicated a high correlation among the selected genes (Figure 3).

### 2.4. Genes Related to Biotic Stress Pathways

#### 2.4.1. Overview of Biotic Stress-Related Pathways

**Figure 4:**
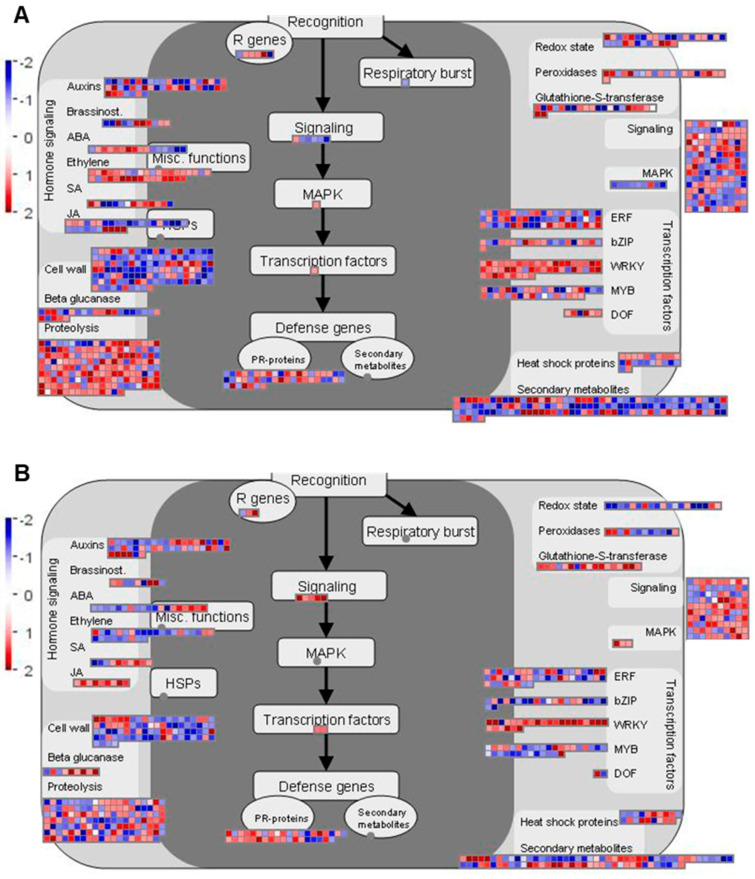
Distribution of differentially expressed genes involved in the biotic stress response in two rutabaga hosts at 7 days after inoculation with *Plasmodiophora brassicae*. (**A**) ‘Wilhemsburger’ and (**B**) ‘Laurentian.’ The log_2_ fold-changes are presented on a scale where red represents upregulation and blue represents downregulation. ABA, abscisic acid; JA, jasmonic acid; SA, salicylic acid; bZIP, basic region-leucine zipper; ERF, APETALA2/Ethylene-responsive element binding protein family; WRKY, WRKY transcription factor; MYB, MYB transcription factor; DOF, DNA-binding one zinc finger transcription factor; MAPK, mitogen-activated protein kinase; PR-protein, pathogenesis-related protein; R genes, resistance genes.

**Figure 5:**
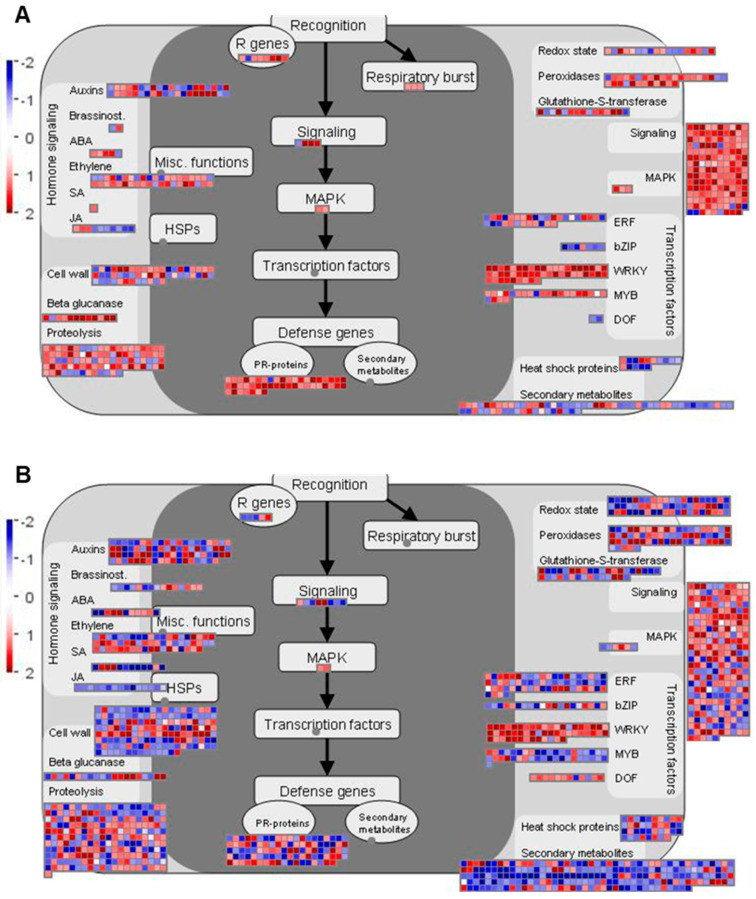
Distribution of differentially expressed genes involved in the biotic stress response in two rutabaga hosts at 14 days after inoculation with *Plasmodiophora brassicae*. (**A**) ‘Wilhemsburger’ and (**B**) ‘Laurentian.’ The log_2_ fold-changes are presented on a scale where red represents upregulation and blue represents downregulation. ABA, abscisic acid; JA, jasmonic acid; SA, salicylic acid; bZIP, basic region-leucine zipper; ERF, APETALA2/Ethylene-responsive element binding protein family; WRKY, WRKY transcription factor; MYB, MYB transcription factor; DOF, DNA-binding one zinc finger transcription factor; MAPK, mitogen-activated protein kinase; PR-protein, pathogenesis-related protein; R genes, resistance genes.

Regulation of various biotic stress-associated responses is important for the host defense against *P. brassicae* infection [26,32,33]. Therefore, we further investigated DEGs related to biotic stress using MapMan [34], to visualize the regulation of genes in major pathways and processes related to this functional category (Figure 4 and Figure 5 and Figure S2).

**Figure 6:**
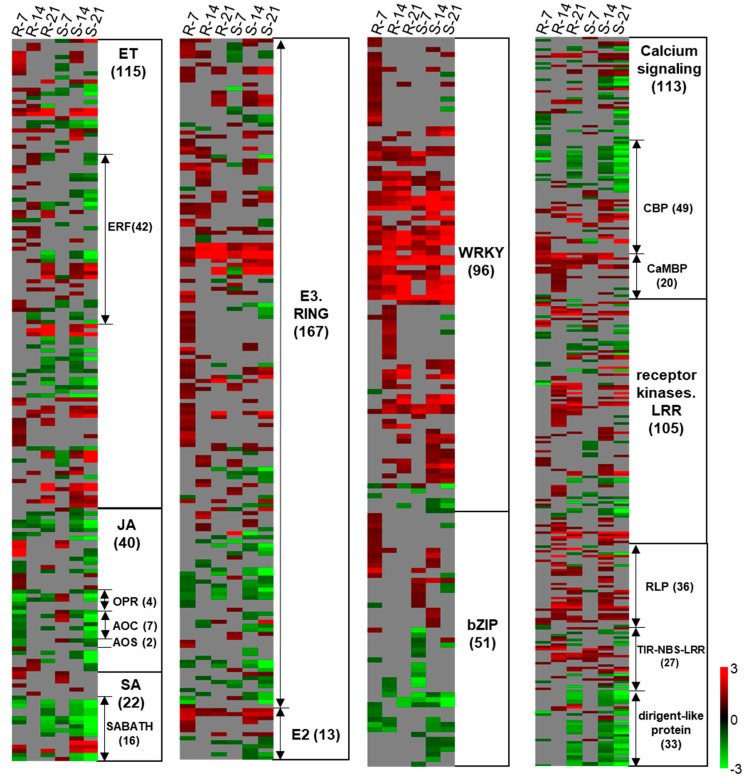
Heatmaps of differentially expressed genes in the resistant (R) rutabaga ‘Wilhelmsburger’ and the susceptible (S) ‘Laurentian’ in response to *Plasmodiophora brassicae* through the time course. Only genes that showed significant differential expression in each host at 7 or 14 dai were selected. In the heatmap scale used in this diagram, red indicates upregulation, green indicates downregulation, and gray indicates no significant regulation. ERF, ethylene response factor; JA, jasmonic acid; OPR, oxophytodienoate reductase; AOC, allene oxide cyclase; AOS, allene oxide synthase; SA, salicylic acid; SABATH, SABATH methyltransferase gene family; CBP, calcium binding protein; CaMBP, calmodulin-binding protein; LRR, leucine-rich repeat; RLP, receptor like protein. The number of transcripts for each term are indicated in parentheses.

At 7 dai, more DEGs assigned to biotic stress were identified in ‘Wilhelmsburger’ than in ‘Laurentian’ (Figure 4). Among the categories designated in MapMan for biotic stress, most DEGs annotated as *WRKY* TFs were upregulated in both hosts. In ‘Wilhelmsburger,’ most of the DEGs in the ET category were upregulated, while these were mostly downregulated in ‘Laurentian.’ Although fewer jasmonic acid (JA)-related DEGs were identified in ‘Laurentian’ than in ‘Wilhelmsburger,’ all DEGs in ‘Laurentian’ were upregulated, while most genes in ‘Wilhelmsburger’ were downregulated. At 14 dai, while fewer DEGs in ‘Wilhelmsburger’ than in ‘Laurentian’ were assigned to biotic stress, most were upregulated (Figure 5). The largest number of DEGs assigned to various biotic stress related categories were identified in both cultivars at 21 dai; most of these genes were downregulated, with few evident differences between cultivars (Figure S2). Collectively, the results suggest that the host DEGs related to biotic stress identified at 7 and 14 dai showed a clearer distinction in their response to clubroot, than genes regulated at 21 dai. Therefore, we further analyzed DEGs involved in some major categories related to biotic stress and concentrated on differences at 7 and 14 dai (Figure 6).

#### 2.4.2. Genes Related to SA, ET, and JA Metabolism

Salicylic acid, JA, and ET are important pathogen-responsive plant hormones. In general, a dichotomy has been established for SA vs. JA/ET in response to biotrophic and necrotrophic pathogens, respectively [35]. This dichotomy is not always clear cut, however, and JA- or ET-related genes have been suggested to be involved in resistance responses to some biotrophic pathogens, including *P. brassicae*, *Plasmopara viticola* and *Botryosphaeria dothidea* [36,37,38,39]. In Arabidopsis, genes involved in both the SA and ET pathways were upregulated at 7 dai during a partially resistant response to *P. brassicae*, while genes involved in the JA pathways were downregulated [39]. A clubroot resistant inbred line of Chinese cabbage (*B. rapa*) carrying the *CRd* gene activated genes related to the JA, ET, and SA signaling pathways after challenge with an avirulent *P. brassicae* pathotype, which was suggested by the authors to reflect a more potent activation of ETI [37]. 

The activation of genes involved in SA-mediated pathways has been reported widely in resistant reactions following *P. brassicae* inoculation [31,37,40,41]. Isochorismate synthase 1 (*ICS1*) and *ICS2* are two genes redundantly involved in SA synthesis [42]. In our study, two transcripts corresponding to *ICS1* (BnaA07g22090D and BnaC06g22820D) were upregulated in both ‘Wilhelmsburger’ and ‘Laurentian’ across the three time-points; one transcript corresponding to gene *ICS2* (BnaC08g18420D) was upregulated in both hosts at 7 and 14 dai (Supplementary Table S1). The marker gene for SA-mediated responses, pathogenesis-related gene 1 (*PR1*, BnaC03g45470D), showed high upregulation in both ‘Laurentian’ and ‘Wilhelmsburger’ over time, with the exception of no significant regulation in ‘Laurentian’ at 7 dai (log_2_ FC = 2.6, *q* value > 0.05) (Supplementary Table S1). Upregulation of these genes suggests the involvement of SA-triggered immunity in both hosts. The same *ICS2* and *PR1* genes were also upregulated in ‘Laurentian’ at 7, 14, and 21 dai, when it was challenged with *P. brassicae* pathotype 5X [31]. In both hosts, most genes related to SA metabolism corresponded to downregulated transcripts belonging to the SABATH methyltransferase gene family (Figure 6). Members of this family are important for the methylation of phytohormones [43], which can inactivate SA by converting it to methyl salicylate [44]. At 7 dai, three of four transcripts belonging to the SABATH methyltransferase gene family were downregulated in ‘Laurentian,’ and seven of nine transcripts of the same family were downregulated in ‘Wilhelmsburger,’ including one that was upregulated in ‘Laurentian’ (*BSMT1*, BnaA03g31730D) (Figure 6 and Supplementary Table S2). The clubroot pathogen can manipulate host SA levels to weaken host defenses, by secreting methyltransferase PbBSMT, which leads to strong conversion of SA to methyl salicylate at infection sites; overexpression of *BSMT1* in Arabidopsis reduced SA levels by half, although this manipulation alone did not alter susceptibility to *P. brassicae* [45]. Our results suggest stronger repression of SA methylation in ‘Wilhelmsburger’ than in ‘Laurentian’ at 7 dai, but a SA-mediated response is likely involved in both cultivars.

Ethylene-mediated responses are part of clubroot defense mechanisms in plants with various backgrounds. For example, genes related to signaling and ET metabolism were upregulated in resistant plants carrying the CR gene *Rcr1* relative to susceptible plants that lacked this gene [46]. Similarly, in a Chinese cabbage inbred line carrying a CR gene *CRd*, ET signaling-related genes were upregulated when challenged with an avirulent pathotype of *P. brassicae*, but were not regulated when challenged with a virulent pathotype [37]. Several Arabidopsis mutants of genes within the ET signaling pathway showed increased susceptibility to *P. brassicae* infection [47]. In our study, regulation of genes involved in the ET category showed the greatest differences between the two cultivars at 7 dai, as 33 of 38 transcripts in ‘Wilhelmsburger’ were upregulated and 23 of 30 significantly regulated transcripts in ‘Laurentian’ were downregulated (Figure 6 and Supplementary Table S2). Ethylene response factors (*ERFs*) are important in activating other defense genes in response to *P. brassicae* [47]. In our study, ‘Wilhelmsburger’ had more upregulated transcripts annotated as *ERFs* (11) than ‘Laurentian’ (2) at 7 dai, a trend that was also observed at 14 dai (Figure 6). For example, at 7 dai, three transcripts corresponding to the ethylene response factor 104 (*ERF104*) were upregulated in ‘Wilhelmsburger,’ and two of those transcripts were downregulated in ‘Laurentian’ (BnaC07g31350D and BnaA03g40380D). *ERF104* is activated by MAP kinase 6 (*MPK6*) upon perception of bacterial flagellin peptide flg22 in Arabidopsis, which alters plant susceptibility to *Pseudomonas syringae* [48]. A transcript matching the gene *MPK6* (BnaC03g24500D) was activated only in ‘Wilhelmsburger’ but not in ‘Laurentian’ at 7 dai (Supplementary Table S1), suggesting that upregulation of *ERF104* and *MPK6* may be involved in clubroot resistance. In addition, three transcripts matching *ERF11* were upregulated in ‘Wilhelmsburger’ and not in ‘Laurentian’ at 7 and/or 14 dai (Table S2). Similarly, overexpression of *ERF11* in apple increased its resistance to the biotrophic fungus *B. dothidea* by enhancing accumulation of SA and expression of SA synthesis-related and signaling-related genes [36], suggesting possible cross-talk between ET and SA in some biotrophic interactions. 

Jasmonic acid-related genes also showed the greatest differences in expression at 7 dai, when 22 of 31 DEGs in ‘Wilhelmsburger’ were downregulated and all nine DEGs in ‘Laurentian’ were upregulated (Figure 6). Six transcripts involved in JA biosynthesis, including 12-oxophytodienoate reductase 1 (*OPR1*, BnaC09g41020D and BnaA10g17650D), allene oxide cyclase 2 (*AOC2*, BnaA06g33410D, BnaC09g52570D, and BnaA09g19550D), and allene oxide synthase (*AOS*, BnaA02g23180D), were downregulated in ‘Wilhelmsburger’ and upregulated in ‘Laurentian’ (Table S2). This contrasting pattern of expression is consistent with the regulation of JA biosynthesis genes in CR and CS responses, which was reported in other studies at early stages of infection [39,49]. At 14 and 21 dai, genes in the JA category showed general downregulation in both hosts (Figure 6). Collectively, our results suggest that JA does not seem central to defense in the resistant cultivar, while it may be a mechanism that is activated in this susceptible interaction. 

#### 2.4.3. Pathogenesis-Related (*PR*) Genes

The most notable differences in regulation of *PR* genes between ‘Wilhelmsburger’ and ‘Laurentian’ were detected at 14 dai. At this time-point, while fewer *PR* genes were identified in the resistant vs. the susceptible host, most were upregulated in the former (46 of 47), while 45 of 98 genes were downregulated in the latter (Figure 5).

Transcripts encoding RLP and TIR-NBS-LRR proteins showed general upregulation in both hosts at 14 dai (Figure 6). All 19 genes encoding RLPs were upregulated in ‘Wilhelmsburger,’ while 20 of 21 were upregulated in ‘Laurentian.’ Receptor-like proteins are key components of PRRs, which recognize PAMPs or endogenous DAMPs to activate PTI-mediated responses [14]. For example, the protein RLP30 is required for perception of a fungal PAMP known as sclerotinia culture filtrate elicitor 1 (SCFE1) [50]. Another protein RLP23 binds to a conserved 20 amino acid fragment from necrosis and ethylene-inducing peptide 1-like proteins (NLPs) produced by multiple bacterial, oomycete, and fungal microbes, and mediates plant resistance to diverse pathogens such as *Phytophthora infestans* and *Sclerotinia sclerotiorum* [51]. In our study, transcripts encoding RLP23 and RLP30 were upregulated in both hosts (Supplementary Table S2), suggesting a role in basal responses to clubroot. In addition, 10 upregulated transcripts corresponding to TIR-NBS-LRR proteins were identified in ‘Wilhelmsburger’ and nine were identified in ‘Laurentian.’ Only four of these, however, were found to be commonly upregulated in the two hosts (Figure 6 and Supplementary Table S2). TIR-NBS-LRR genes are *R* genes linked to ETI responses [13] and are important for clubroot resistance, representing one of the main sources of candidate CR genes [20,21]. We identified the gene BnaA03g29300D, which is the homolog of the CR gene, *CRd* (Bra001175), in *B. rapa* [21]. This gene was upregulated earlier in ‘Wilhelmsburger’ (14 dai) than in ‘Laurentian’ (21 dai). Another gene, BnaAnng17440D, which was upregulated in ‘Wilhelmsburger’ but not regulated in ‘Laurentian’ at 7 dai, was similar to the candidate resistance gene *CRd* (Bra001160) [21], based on our sequence alignment analysis. Resistance associated with *CRd* is related to the activation of genes involved in both SA and ET signaling pathways [37], which is consistent with the upregulation of genes related to these hormones in this study.

In addition, *PR* genes involved in SA-mediated defense also showed differential regulation between the two hosts at 7 dai, including *PR1,* which was only upregulated in ‘Wilhelmsburger,’ and the nonexpresser of *PR* genes 1 (*NPR1*)-like protein 3 (*NPR3*), which was upregulated only in ‘Laurentian’ (Supplementary Table S1). The upregulation of *PR1* in clubroot resistance responses has been reported widely [24,25,37]. *PR1* is a marker gene for SA-mediated resistance, which is positively regulated by TGACG motif-binding protein (*TGA*) and *NPR1* genes [52]. In contrast, *NPR3* is a co-repressor of SA-induced defense gene expression; it interacts with *TGAs* to inhibit expression of defense-related genes under low SA levels, while its repression is inhibited when SA is high [53].

Expression of transcripts belonging to the dirigent-like protein family was most divergent between ‘Laurentian’ and ‘Wilhelmsburger’ at 14 dai. Twenty-seven of these transcripts were downregulated in ‘Laurentian’ at this time, while two were upregulated in ‘Wilhelmsburger’ (Figure 6). Genes belonging to this family are thought to participate in biotic and abiotic defense by increasing lignan and lignin synthesis [54]. Lignin synthesis positively regulates clubroot resistance [55,56]. Our results showed that, at 14 dai, more genes involved in lignin biosynthesis were downregulated in ‘Laurentian’ (23 of 26 genes) than in ‘Wilhelmsburger’ (four of eight genes) (Supplementary Figure S3). At this time-point, two transcripts matching genes encoding dirigent protein 6 (*DIR6*, BnaAnng27090D and BnaC01g15510D) were downregulated in ‘Laurentian,’ but were not regulated in ‘Wilhelmsburger.’ These genes contain the TIR-NBS-LRR domain, and their sequences showed high similarity to Bo7g109000 in *B. oleracea*, a gene that is located in the target region of a major clubroot resistance gene *Rcr7* [57]. This indicates that greater downregulation of genes in the dirigent-like protein family in ‘Laurentian’ may be associated with more rapid galling of the roots.

#### 2.4.4. Signaling

Signaling networks are important for the activation of plant defenses against clubroot [33]. As with the *PR* genes, genes involved in signaling pathways showed notable differences in expression at 14 dai, with a greater proportion of these genes upregulated in ‘Wilhelmsburger’ vs. ‘Laurentian’ (Figure 5). This was especially evident for calcium regulated genes and LRR receptor kinases (Figure 6). A transcript encoding the LRR receptor kinase pep 1 receptor 2 (PEPR2, BnaC05g49970D) was upregulated in ‘Wilhelmsburger’ at 7 dai but downregulated in ‘Laurentian’ at 7 and 14 dai (Supplementary Table S2). The protein PEPR2 perceives Arabidopsis DAMP Pep1/2 peptide, and cooperates with ET to amplify resistance to *Botrytis cinerea* [58,59]. In addition, three transcripts encoding a protein suppressor of BIR1–1 (*SOBIR1*) were upregulated in ‘Wilhelmsburger’ at 14 dai, of which only one was upregulated in ‘Laurentian.’ The protein SOBIR1 interacts with various RLPs, such as RLP23 and RLP30 (discussed above), to enhance plant immunity upon fungal pathogen challenge [50,51,60].

In the calcium signaling subcategory, most upregulated transcripts in ‘Wilhelmsburger’ at 14 dai encoded calcium binding proteins (CBPs; all eight transcripts upregulated) and calmodulin-binding proteins (CaMBPs; all 17 transcripts upregulated). At the same time-point, a large portion of *CBP* transcripts (14 of 26) was downregulated in ‘Laurentian,’ and only seven transcripts encoding CaMBPs were upregulated in this cultivar (Figure 6). This is consistent with previous transcriptomics studies suggesting a Ca^2+^ influx in the clubroot resistance response [24,25]. Several members of the CaMBP family are important in plant defense. For example, CaMBP 60-like G (*CBP60g*) and the closely related SAR deficient 1 (*SARD1*) gene are involved in SA biosynthesis and pathogen defense responses [44]. These two genes showed upregulation in the resistant interaction at 14 dai when challenged with *P. brassicae* pathotype 5X [31]. Our results showed that at 14 dai, all three transcripts matching *CBP60g* were upregulated only in ‘Wilhelmsburger.’ At that same time, five transcripts annotated as *SARD1* were also upregulated in ‘Wilhelmsburger,’ of which three were upregulated in ‘Laurentian’ (Supplementary Table S2). Ca^2+^ also activates burst oxidase homolog (RBOH) proteins, which are key factors in enhancing production of ROS during the plant immunity response [61]. This type of response has been well studied in clubroot interactions [24,33,41,49,62,63]. In our analysis, three transcripts annotated as *RBOHs* (*RBOHA*, *RBOHC,* and *RBOHG*) were upregulated in ‘Wilhelmsburger’ but not in ‘Laurentian’ at 14 dai (Supplementary Table S1). The homologs of the same three genes were upregulated in clubroot resistant wild cabbage (*B. macrocarpa*) following *P. brassicae* infection [62]. Collectively, our results support activation of genes involved in calcium-dependent defense responses against clubroot.

#### 2.4.5. Transcription Factors

Transcription factors play important roles in modulating the host immune responses [64]. The activation of *WRKY* TFs in plants in response to *P. brassicae* has been reported widely [24,25,37,39,41,63]. In the present study, most *WRKY* TFs were upregulated in both hosts over the entire time-course (Figure 4 and Figure 5 and Supplementary Figure S2). Some transcripts were upregulated in ‘Wilhelmsburger’ but not regulated in ‘Laurentian’ at 7 or 14 dai, including *WRKY22*, *WRKY29*, *WRKY33,* and *WRKY46* at 7 dai, and *WRKY46* and *WRKY53* at 14 dai. *WRKY46*, *WRKY53*, and *WRKY70* are involved in the SA-signaling pathway and play overlapping and synergistic roles in plant resistance to *P. syringae* [65]. In our study, at least five transcripts annotated as *WRKY70* genes were upregulated in both hosts at all three time-points, except in ‘Laurentian’ at 7 dai (three genes). These results suggest that the activation of *WRKY46*, *WRKY53*, and *WRKY70* is associated with SA-mediated defense responses to clubroot, and that regulation of *WRKY46* and *WRKY70* at 7 dai may be related to enhanced SA-mediated responses in ‘Wilhelmsburger.’ *WRKY22* and *WRKY29* are activated in PTI and regulate resistance to *P. syringae* and *B. cinerea* [66]. While *WRKY33* typically has been associated with resistance to necrotrophic fungal pathogens [67], its upregulation in response to *P. brassicae* has also been reported and is believed to be modulated by *MPK6* [25,41,63]. In our study, a transcript matching *MPK6* and another matching the ET synthesis gene 1-amino-cyclopropane-1-carboxylate synthase 2 (*ACS2*), which is activated by *MPK6*-*WKRY33* [68], exhibited an expression pattern similar to *WRKY33* in both ‘Wilhelmsburger’ and ‘Laurentian’ at 7 dai (Supplementary Table S2). This suggests that *MPK6*-*WKRY33* may contribute to ET synthesis to enhance clubroot resistance. In addition to *WRKY33*, *WRKY22*, *WRKY29*, and *WRKY46* are also activated by *MPK6* following pathogen challenge [66,68,69]. For example, in cabbage showing resistance to *P. brassicae,* the activation of *MEKK1*-*MKK4*/*MKK5*-*MPK3*/MPK6 resulted in upregulation of *WRKY22*/*WRKY29*/*WRKY33* [63]. Collectively, these results indicate a central role of *WRKYs* in regulatory defense responses to clubroot.

Members of the basic leucine zipper (*bZIP*) TF family are important regulators of many key developmental and physiological processes, including biotic stress responses [70]. The upregulation of some *bZIPs* has been associated with CR responses [39,41]. *TGAs*, a type of *bZIP* TF, are important for activating SA-regulated genes such as *PR1* [52]. In our study, ‘Wilhelmsburger’ showed a higher proportion of upregulated *bZIP* TFs than ‘Laurentian’ at 7 dai (Figure 6). Three transcripts annotated as *TGA10* and five encoding *TGA1* were upregulated only in ‘Wilhelmsburger’ at 7 dai (Supplementary Table S2), which may be associated with the upregulation of *PR1* observed in this host. 

#### 2.4.6. Protein Degradation

Proteolysis-related genes also showed distinct differences in expression between ‘Laurentian’ and ‘Wilhelmsburger.’ While numerous genes involved in protein degradation were regulated in both hosts, proportionally more genes were upregulated in ‘Wilhelmsburger’ and downregulated in ‘Laurentian’ at 7 and 14 dai (Figure 5 and Figure 6). At 7 dai, five transcripts encoding E2 ubiquitin-conjugating enzymes (E2) and 57 transcripts encoding E3 ubiquitin ligase (E3) RING proteins were upregulated only in ‘Wilhelmsburger’ (Figure 6), of which two WAV3 homolog 1 (*WAVH1*) genes (BnaC04g35190D and BnaA04g13100D) and two BCA2Â zinc finger ATL 10 (*BTL10*) genes (BnaA06g17960D and BnaCnng37520D) were downregulated in ‘Laurentian’ (Supplementary Table S2). At 14 dai, proportionally more transcripts encoding E2 and E3 RING proteins were upregulated in ‘Wilhelmsburger’ (38 of 50 transcripts) than in ‘Laurentian’ (49 of 82 transcripts) (Figure 6). The E2 ubiquitin-conjugating enzymes and E3 ubiquitin ligase proteins are key components of the ubiquitin–proteasome system. These enzymes bind to ubiquitin and form multimers that attach to proteins, targeting them for degradation by 26S proteasomes. This ubiquitin–proteasome system interacts with key components of plant immunity to positively or negatively regulate resistance to plant pathogens [71]. Genes encoding RING proteins in the E3 ubiquitin pathway were upregulated in *Rcr1*-mediated clubroot resistance and downregulated in susceptible Arabidopsis following *P. brassicae* infection [32,72]. Several genes in the Arabidopsis Tóxicos en Levadura (*ATL*) family encoding E3 RING proteins are involved in plant defense against pathogens [71]. In our study, two transcripts matching *ATL2* and three transcripts matching *ATL31* were upregulated in ‘Wilhelmsburger’ at both 7 and 14 dai, of which one of each were also upregulated in ‘Laurentian’ at 14 dai. (Supplementary Table S2). *ATL2* and *ATL31* are induced by pathogens or PAMPs [73]. The expression of *PR1* was induced in Arabidopsis mutants constitutively expressing *ATL2* [74]. Overexpression of *ATL31* in Arabidopsis increased resistance to *P. syringae*, while knock-out of these genes decreased resistance [75]. One of our *ATL31* genes (BnaA09g03720D) was upregulated in the resistant ‘Laurentian’ but downregulated in susceptible ‘Brutor’ (*B. napus*) when inoculated with *P. brassicae* pathotype 5X [31], suggesting that SA levels may increase in the resistant interaction.

### 2.5. Analysis of Genes with Opposite Regulation in the Resistant vs. Susceptible Hosts

We identified many genes with opposite patterns of regulation in the two hosts at each of 7, 14, and 21 dai (Figure 2B–D), and discussed some of these genes in the Section 2.4. Here, we further investigated their expression and putative functions to select good candidates for gene editing-based functional validation.

**Figure 7:**
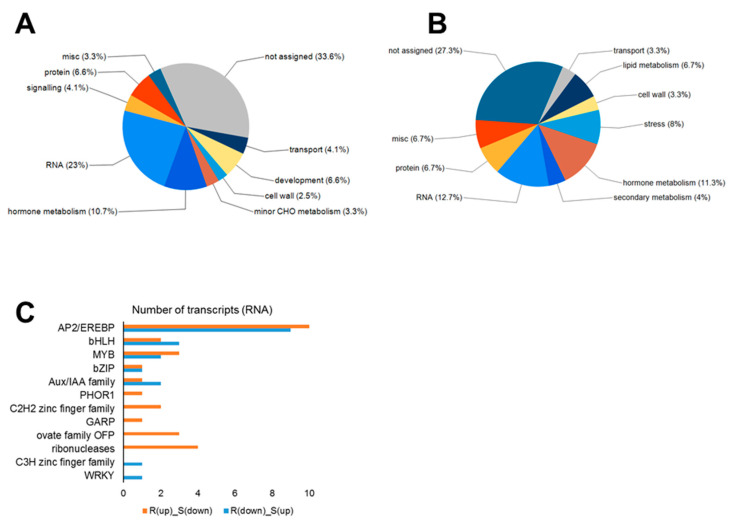
Distribution of the number of transcripts showing opposite regulation patterns in the resistant (R) rutabaga ‘Wilhelmsburger’ and the susceptible (S) ‘Laurentian’ at 7 days after inoculation with *Plasmodiophora brassicae*, using Mapman annotation. (**A**) Pie chart generated from transcripts upregulated in ‘Wilhlemsburger’ and downregulated in ‘Laurentian.’ **(B**) Pie chart generated from transcripts downregulated in ‘Wilhelmsburger’ and upregulated in ‘Laurentian.’ (**C**) Number of transcripts related to RNA regulation.

We first divided genes identified at 7 dai into two lists: genes upregulated in ‘Wilhelmsburger’ but downregulated in ‘Laurentian’ (List A and Supplementary Table S3a) and genes downregulated in ‘Wilhelmsburger’ but upregulated in ‘Laurentian’ (List B and Supplementary Table S3b). Genes on each list were then grouped based their functional categories in Mapman [34]. The majority of genes on both lists (33.6% in List A and 27.3% in List B) belonged to the “not assigned” category (i.e., did not match any Mapman classification), followed by “RNA” (23% in List A and 12.7% in List B) and “hormone metabolism” (10.7% in List A and 11.3% in List B) (Figure 7A,B). These results supported the importance of transcriptional regulation and hormone metabolism in the *B. napus*-*P. brassicae* interaction at 7 dai. The functional category “lipid metabolism” was identified only on List B (6.7%). Five of 10 transcripts involved in lipid metabolism were related to lipid synthesis (Supplementary Table S3b), which is consistent with the upregulation of lipid synthesis genes in *P. brassicae*-infected roots and the accumulation of lipid droplets in the parasite as a nutrient sink for *P. brassicae* survival [76,77]. In addition, JA is a lipid-derived signal [78,79], consistent with the similar regulation patterns of JA and lipid synthesis related genes in this study. A transcript matching gene fatty acid desaturase 7 (*FAD7*, BnaA03g31600D) was downregulated in ‘Wilhelmsburger’ (log_2_FC = −1.21) and upregulated in ‘Laurentian’ (log_2_FC = 1.02). Fatty acid desaturase 7 (*FAD7*) is involved in the synthesis of both fatty acid and JA, but it inhibits SA accumulation and signaling [80], suggesting that this gene could be an important candidate susceptibility factor.

Most transcripts assigned to the “RNA” functional category matched the APETALA2 (*AP2*)/ethylene-responsive element binding protein (*EREBP*) (10 on List A and 9 on List B) (Figure 7C). Members of the *ERF* subfamily of AP2 TFs are involved in the regulation of disease resistance pathways, and some *ERFs* have been shown to be regulated by plant hormones (ET, JA, and SA) and pathogen challenge [81]. The high proportion of additional regulated *ERFs* in the AP2/EREBP gene family in ‘Wilhelmsburger’ and ‘Laurentian’ at 7 dai (Supplementary Tables S3a,b) supports the importance of *ERFs* in the host response to *P. brassicae*. However, *ERFs* may play different roles in resistant and susceptible interactions, since they are distributed on both List A and List B. In addition, two of the transcripts on List A matched a TF *MYB15*, which is consistent with upregulation of this gene in a CR rapeseed accession but not in a CS accession upon *P. brassicae* infection [82]. *MYB15* contributed to resistance to *P. syringae* in Arabidopsis [83]. In Chinese wild grape (*Vitis quinquangularis*), *MYB15* was induced when plants were treated with flg22 and *P. viticola*, making its promoter a potential target for disease resistance breeding [84]. This suggests that *MYB15* may also be a good candidate for functional validation in clubroot resistance.

On List A, the transcript showing the most distinct regulation in the two hosts at 7 dai matched the gene plastidic type I signal peptidase 2A (*PLSP2A*, BnaC05g04750D); this transcript showed the greatest upregulation in ‘Wilhelmsburger’ (log_2_FC = 4.86) and the greatest downregulation in ‘Laurentian’ (log_2_FC = −3.52). *PLSP2A* corresponds to a thylakoidal processing peptidase usually expressed in both photosynthetic tissues and roots and is important for thylakoid membrane organization [85]. In cabbage (*B. oleracea*), a large portion of differentially modulated proteins in resistant vs. susceptible interactions with *P. brassicae* was localized to the thylakoid [86]. These results suggest that *PLSP2A* could be an important candidate for resistance to *P. brassicae*. A gene involved in phosphorylation of the thylakoid membrane has been suggested as a candidate for resistance to *Leptosphaeria maculans* in *B. napus* [87]. On list B, the two transcripts showing the greatest downregulation in ‘Wilhelmsburger’ matched two copies of “cytochrome P450, family 94, subfamily C, polypeptide 1” (CYP94C1, BnaC04g16670D, and BnaA07g13320D, log_2_FC = ~−4), both of which were upregulated in ‘Laurentian’ (log_2_FC = ~1.7). In addition, transcripts matching three copies of *CYP94B1* (BnaC03g50910D, BnaA06g38770D, and BnaA09g06580D) were also identified on List B. Both *CYP94C1* and *CYP94B1* are induced by JA treatment and involved in the catabolism and deactivation of jasmonoyl-L-isoleucine (JA-Ile), a major bioactive form of JA [88]. Expression of *CYP94C1* also increased in early galling tissues in Chinese sumac (*Rhus javanica*) infested by aphids (*Schlechtendalia chinensis*) [89]. Collectively, these findings suggest that *CYP94C1* and *CYP94B1* could be good candidate susceptibility factors during clubroot development.

Transcripts showing opposite regulation patterns in ‘Wilhelmsburger’ and ‘Laurentian’ at 14 dai are listed in Table S3c,d. At this time-point, the transcript showing the greatest upregulation in ‘Wilhelmsburger’ corresponded to an LRR transmembrane protein kinase (BnaC05g27810D) (log_2_FC = 4.12), which was downregulated in ‘Laurentian’ (log_2_FC = −1.25). Its orthologous gene in Arabidopsis encodes a protein localized to the plasma membrane, where a large portion of upregulated gene products were identified in a clubroot resistant reaction in *B. rapa* [46]. Considering the possible roles of LRR protein kinases in mediating resistance to pathogens, this gene may be another candidate of resistance. A transcript matching cytochrome p450 79f1 (*CYP79F1*) was downregulated in ‘Laurentian’ (log_2_FC = −2.52) and upregulated in ‘Wilhemsburger’ (log_2_FC = 1.15). An Arabidopsis mutant of *CYP79F1* had reduced aliphatic glucosinolate and increased indole glucosinolate content [90]. Higher aliphatic glucosinolates and lower indole glucosinolates levels have been associated with clubroot resistance in previous studies [41,91]. Transcripts matching two other key genes involved in aliphatic glucosinolate synthesis (*CYP83A1* and bile acid transporter 5 (*BAT5*)) [92,93] showed a pattern of regulation similar to *CYP79F1*. Recently, *CYP83A1* has been screened as a candidate gene for clubroot resistance in rapeseed, by combining functional enrichment analysis, co-expression network analysis, and haplotype analysis [82]. These results suggest that these aliphatic glucosinolates synthesis-related genes may be good candidates for increasing clubroot resistance.

At 21 dai, the most upregulated transcript in ‘Wilhelmsburger’ and the most downregulated transcript in ‘Laurentian’ did not match any *B. napus* or Arabidopsis gene annotations. A transcript matching BnaA04g25230D/AT2G43610 belonging to the chitinase family protein, however, showed the second highest level of upregulation in ‘Wilhelmsburger’ (log_2_FC = 1.53), contrasting with downregulation in ‘Laurentian’ (log_2_FC = −1.53) (Supplementary Table S3e). Chitinases are a subgroup of PR proteins which attack pathogens directly by hydrolyzing chitin, a component of *P. brassicae* and many fungal cell walls [94,95]. The differential regulation of chitinase genes has been described in the defense response to *P. brassicae* [24,56,96]. 

### 2.6. A Model of the Molecular Response in the Resistant Cultivar ‘Wilhemsburger’ to P. brassicae

**Figure 8:**
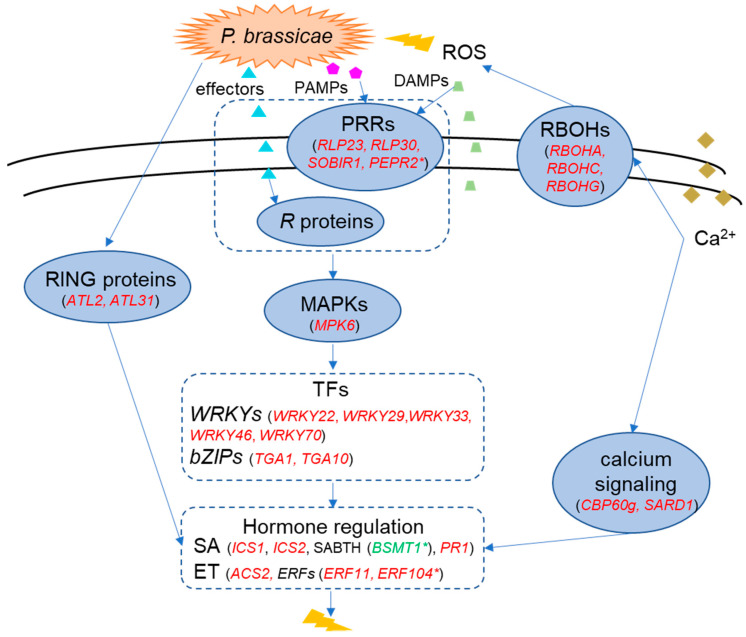
Model illustrating the major networks of the resistance response in the rutabaga ‘Wilhelmsburger’ to *Plasmodiophora brassicae* pathotype 3A. Important genes in each functional category are indicated in parentheses. Genes in red are upregulated, while those in green are downregulated in the resistant cultivar ‘Wilhelmsburger.’ Genes marked with an asterisk (*) were inversely regulated in the susceptible cultivar ‘Laurentian.’ Lightning bolt symbols indicate defense responses.

Based on the discussion above, we propose a model of the major defense mechanisms induced by *P. brassicae* pathotype 3A in its interaction with the resistant *B. napus* ‘Wilhelmsburger’ (Figure 8). Upon infection, PRRs (e.g., *RLP23*, *RLP30*, *SOBIR1*, *PEPR2*) on the host cell surface recognize extracellular PAMPs and DAMPs, leading to PTI. In parallel, *R* proteins (e.g., *TIR-NBS-LRR*) recognize specific effectors from the pathogen, triggering ETI. The two-layer immunity of PTI and ETI have overlapping roles in the defense network, such as activating MAPKs [97]. Activated MAPKs can phosphorylate TFs to enhance their transcriptional activity [98]. For example, regulation of *MPK6* resulted in the activation of multiple *WRKY* TFs, including *WRKY22*, *WRKY29*, *WRKY33*, and *WRKY46*, in the resistant host in our study. Furthermore, some WRKY TFs may mediate resistance by regulating plant hormone metabolism. *WRKY33* activates the ET biosynthesis gene *ACS2*, while *WRKY46*, *WRKY53*, WKRY70, and some *bZIP* TFs (e.g., *TGA1* and *TGA10*) positively regulate SA signaling. In parallel, *ERF11* and *ERF104* are involved in ET signaling. Our results also suggest the activation of calcium-dependent defenses, which may contribute to the activation of *RBOHs*, key genes for ROS production, and several SA synthesis genes. Infection by *P. brassicae* may also induce expression of RING-type ubiquitin ligase genes. In particular, *ATL2* and *ATL31* may play a role in defense by enhancing SA-mediated responses. The involvement of genes related to SA-mediated responses and their antagonistic effect on JA-related genes as a clubroot defense mechanism is consistent with a recent report by Galindo-González et al. (2020) [31]. Our results suggesting the importance of ET-related genes, however, contrast with the findings of Galindo-González et al. (2020) [31]. This apparent contradiction may reflect specific pathotype by host interactions, and the evaluation of multiple pathotypes with similar hosts may help to identify common defense and susceptibility genes across the clubroot pathosystem. 

In conclusion, our study described genome-wide molecular responses of two rutabaga cultivars following inoculation with a widespread *P. brassicae* pathotype (3A) in western Canada. Our results provide insights into possible common defense responses in both cultivars, as well as cultivar-specific responses. Furthermore, we identified key defense genes that may be further validated using approaches such as gene editing to increase clubroot resistance. Ultimately, an improved understanding of *P. brassicae*/Brassica interactions will aid in the development of novel strategies for clubroot resistance breeding.

## 3. Materials and Methods

### 3.1. Pathogen Material

*Plasmodiophora brassicae* field isolate F3–14, originally collected from the CR canola ‘L135C’ and classified as pathotype 3A on the Canadian Clubroot Differential set [11], was used as the inoculum for this study. The isolate was stored as frozen (−20 °C) root galls and resting spore suspensions were prepared following Strelkov et al. (2006). Briefly, 100 g of the root galls were homogenized in 1 L distilled water (dH_2_O) in a blender for 2 min, with the resulting homogenate filtered through eight layers of cheesecloth to remove any debris. The spore concentration was estimated with a hemocytometer and adjusted to about 1 × 10^7^ spores/mL with dH_2_O.

### 3.2. Plant Material and Inoculation

All experiments were conducted with the rutabagas ‘Wilhelmsburger’ and ‘Laurentian’ (Bejo Seeds Inc., Oceano, CA, USA), which are resistant and susceptible, respectively, to pathotype 3A of *P. brassicae* [11]. The universally susceptible Chinese cabbage (*Brassica rapa* L. var. *pekinensis*) ‘Granaat’ (Bejo Seeds Inc., Oceano, CA, USA) was also included as a check in all inoculations, to ensure that the inoculum was viable and conditions were favorable for clubroot development. Eight-day-old seedlings, germinated in Petri dishes on moistened filter paper, were inoculated by the root dip method following Strelkov et al. (2006) [99]. The seedlings were briefly (10 s) dipped in the resting spore suspension and planted in pots (6 cm × 6 cm × 6 cm) filled with water-saturated Sunshine LA4 potting mix (SunGro Horticulture, Vancouver, BC, Canada). An additional 1 mL of inoculum was added to the base of each seedling with a micropipette to ensure strong disease pressure. Non-inoculated control plants were transferred directly from the Petri dishes to the potting mix. Plants were placed in insect cages (47.5 cm × 47.5 cm × 93.0 cm) to avoid potential insect infestations that could interfere with plant responses, and the experiment was conducted in a greenhouse under long day conditions (16 h) at 22 °C. Roots were harvested at 7, 14, and 21 dai, washed with tap water and briefly dried on paper towels before being collected in Falcon tubes (Thermo Fisher Scientific, Waltham, MA, USA) and flash-frozen in liquid nitrogen. Five independent biological replicates were assigned for each treatment, with 27 pooled plants in each biological replicate. Clubroot symptom severity was evaluated at 45 dai on a 0–3 scale following Kuginuki et al. (1999), where 0 = no visible galls, 1 = a few small galls, 2 = moderate galling, and 3 = severe galling [100]. Five independent biological replicates, with 30 plants per replicate, were used to rate symptom severity. Then, the severity rating results were used to calculate a DI using the formula of Horiuchi and Hori (1980) [101] as modified by Strelkov et al. (2006): DI (%) = [(*n*_1_ × 1 + *n*_2_ × 2 + *n*_3_ × 3)/(*n* × 3)] × 100, where *n_1_*, *n_2_*, and *n_3_* refer to the number of plants in each symptom severity class and *n* refers to the total number of plants tested.

### 3.3. RNA Extraction

RNA was extracted from whole-root tissues of each host genotype at each time-point. Pooled tissues of all 27 plants of each biological replicate were ground to a fine powder in a mortar with a pestle in the presence of liquid nitrogen. The RNA was extracted from the 0.1 mL tissue homogenates using 1 mL Trizol (Ambion-Life Technologies, Carlsbad, CA, USA), 0.2 mL chloroform (Fisher Chemical, Fair Lawn, NJ, USA), and precipitated with 0.5 mL 2-propanol (Fisher Chemical, Fair Lawn, NJ, USA), followed by a cleanup step using RNeasy Mini Kit (Qiagen, Hilden, Germany) according to the manufacturer’s instructions. The RNA was treated with DNAse (Qiagen, Hilden, Germany) for 15 min at room temperature to remove any DNA contamination, and the quantity, purity, and quality of the RNA were assessed with a NanoDrop 2000c Spectrophotometer (Thermo Fisher Scientific, Waltham, MA, USA) and the Agilent 2200 TapeStation system (Agilent, Santa Clara, CA, USA).

### 3.4. RNA-Seq Analysis

Three RNA samples (biological replicates) per treatment with RNA Integrity Numbers (RIN) ≥ 8.0 were sent to Oklahoma State Genomics for library preparation and sequencing. Library preparation was performed using the KAPA mRNA HyperPrep Kit (KAPA Biosystems, Wilmington, MA, USA) following the manufacturer’s instructions. Products were sequenced using a NextSeq 500 system (Illumina, San Diego, CA, USA) to generate 75-bp single-end reads. Reads were filtered with Trimmomatic [102] to remove low quality reads (phred score < 33), adapters, leading/trailing low quality or unknown bases, and reads shorter than 36 bases. The quality of the filtered reads was checked using fastqc (http://www.bioinformatics.babraham.ac.uk/projects/fastqc/) and multiqc [103] prior to further analysis. The sequencing reads were deposited in the NCBI Sequence Read Archive (SRA) under accession number PRJNA641167.

Filtered reads from each fastq file were aligned to the *B. napus* reference genome (AST_PRJEB5043_v1) [104] using Tophat v. 2.11 [105]. Files of the mapped reads and reference genome were used as input for Cufflinks v. 2.2.1 [105], to detect differentially expressed transcripts between inoculated and non-inoculated samples. The Cufflinks analysis was performed with the option of GTF-guide and fragment bias correction using the downloaded reference genome structural annotation; the multi-readcorrection option was also used to weigh read mapping to various genomic locations more accurately. The resulting assembly files from all treatments and biological replicates were merged with Cuffmerge. The number of transcripts per sample was quantified using Cuffquant with the merged consensus transcripts file as a reference. Finally, differentially expressed transcript levels between inoculated and non-inoculated plants at each of the three time-points were detected with Cuffdiff. Expression levels were measured and normalized as reads per kb of transcript per million mapped reads (RPKM). Changes in expression with a log_2_FC > 1 or log_2_FC < −1 and false discovery rate (Benjamini-Hockberg-corrected *q*-value) < 0.05 were considered significant. When calculating log_2_FC, a pseudo-count of RPKM (0.5) was added to each value to decrease the noise from genes with zero or very low expression.

### 3.5. Validation of RNA-Seq Data by qRT-PCR

To validate differential gene expression identified via RNA-seq, qRT-PCR analysis was performed on 10 genes across all treatments and samples (Table S4). These selected genes showed significant expression changes in RNA-seq in at least four of six comparison sets of inoculated vs. non-inoculated samples. Four biological replicates per treatment and time-point were used for cDNA synthesis. Oligo dT (18) (Thermo Fisher Scientific, Waltham, MA, USA)-primed cDNA was synthesized from 500 ng of total RNA using the RevertAid H Minus Reverse transcriptase (Thermo Fisher Scientific, Waltham, MA, USA) according to the manufacturer’s protocol. The absence of genomic DNA contamination was confirmed by end-point PCR, using a 20 μL reaction vol. with 2.5 ng of cDNA, 0.2 mM of each dNTP, 1× Buffer + KCl, 2.5 mM MgCl_2_, 1 unit of high fidelity Taq polymerase, and 0.2 μM of each forward and reverse primer of a clathrin adaptor complex (*CAC)* gene (Table S4). PCR analysis was performed with an initial denaturation step of 3 min at 95 °C followed by 35 cycles of 30 sec at 95 °C, 30 sec at 60 °C, and 1 min at 72 °C, ending with an extension of 10 min at 72 °C. Amplified products were subjected to agarose gel electrophoresis, which resulted in two distinct bands of 125 bp (for cDNA) and 288 bp (for the control genomic DNA).

Quantitative real-time PCR was performed on a ViiA 7 Real-Time PCR System (Applied Biosystems-Life Technologies, Waltham, MA, USA). Each reaction consisted of 5 μL of in-house SYBR-green, 2.5 μL of cDNA (0.25 ng/μL), and 2.5 μL of paired primers (3.2 μM). Reaction conditions included a denaturation step at 95 °C for 5 min followed by 40 cycles at 95 °C for 30 sec and 60 °C for 1 min; melting curves were generated using a cycle of 15 sec at 95 °C, 1 min at 60 °C, and 15 sec at 95 °C. All qRT-PCR assays were conducted with four biological replicates and three technical replicates per biological replicate.

Fold changes between *P. brassicae*-inoculated samples and non-inoculated samples were calculated using the 2^(−ΔΔCt)^ method [106]. To select suitable housekeeping genes for normalization, primers from six previously published housekeeping genes were tested [107,108,109]: *CAC*, guanosine nucleotide diphosphate dissociation inhibitor 1 (*GDI1*), ubiquitin conjugating enzyme 9 (*UBC9*), ubiquitin conjugating enzyme 11 (*UBC11*), tubulin alpha-5 (*TUA5*), and vacuolar ATP synthase subunit E1 (*VHA-E1*).The stability of the genes across all samples was determined with Bestkeeper [110]. The most stable housekeeping genes were *GDI1*, *UBC9*, and *TUA5*. Relative expression of the target genes was quantified using the geometric mean of the cycle threshold (Ct) values of the three selected housekeeping genes (Supplementary Table S4). To compare results obtained from RNA-seq and qRT-PCR analysis, Pearson correlations of log_2_FC values were obtained from the two methods for each combination of treatment and time-point. 

### 3.6. Bioinformatic Analyses

Transcripts were annotated using BLASTX (E value ≤ 1 × 10^−10^) against the *B. napus* [104] and Arabidopsis (*Arabidopsis thaliana*) (TAIR10) [111] databases. Venn diagrams of DEGs were generated using the online tool jvenn (http://jvenn.toulouse.inra.fr/app/example.html) [112]. Principal component analysis of all samples was performed with the ‘ggplot2′ package in R.

MAPMAN [34] was used to display gene sets onto diagrams of metabolic pathways or other relevant processes. The *B. napus* gene IDs matching differentially expressed transcripts in each cultivar at each time-point were used as the input gene list and the gene IDs from the reference genome were used as the background reference. Multi-Experiment Viewer (MeV4.9) [113] was used to visualize log_2_FC of selected genes in both hosts through a time course. The distribution of genes showing opposite regulation patterns between the two cultivars was displayed using FunRich [114].

## Supplementary Materials

Supplementary materials can be found at https://www.mdpi.com/1422-0067/21/21/8381/s1. Figure S1. PCA plots of variation between inoculated and control samples. (A) The PCA plot of ‘Wilhelmsburger’; (B) The PCA plot of ‘Laurentian’. Each condition has three biological replicates. Figure S2. Distribution of DEGs involved in the biotic stress response in the two hosts at 21 dai. (A) ‘Wilhemsburger’ and (B) ‘Laurentian’. Values of log2 fold change for each gene are represented in a scale where red represents upregulation and blue represents downregulation. ABA, abscisic acid; JA, jasmonic acid; SA, salicylic acid; bZIP, basic region-leucine zipper; ERF, APETALA2/Ethylene-responsive element binding protein family; WRKY, WRKY transcription factor; MYB, MYB transcription factor; DOF, DNA-binding one zinc finger transcription factor; MAPK, mitogen-activated protein kinase; PR-protein, pathogenesis-related protein; R genes, resistance genes; Figure S3. Distribution of DEGs involved in lignin synthesis in the two hosts at 14 dai. (A) ‘Wilhemsburger’ and (B) ‘Laurentian’. Values of log2 fold change for each gene are shown in a scale where red represents upregulation and blue represents downregulation. PAL, phenyl alanine ammonia-lyase; 4CL, 4-coumarate:CoA ligase; HCT, hydroxycinnamoyl-CoA shikimate/quinate hydroxycinnamoyl transferase; CCR1, cinnamyl-coenzyme A reductase; F5H, ferulate 5-hydroxylase; COMT, O-methyltransferase; CCOAMT, caffeoyl-CoA 3-O-methyltransferase; CAD, cinnamyl alcohol dehydrogenase. Table S1. Transcripts showing significant expression changes in at least one of six comparison sets of inoculated vs. non-inoculated samples. Table S2. Expression changes and annotations of transcripts matched to Figure 6. Table S3. Transcripts showing opposite regulation patterns in ‘Wilhelmsburger’ and ‘Laurentian’ at 7, 14 and 21 dai. Table S4. Genes used for PCR and qRT-PCR.

## Abbreviations

ABA	Abscisic acid
ACS2	1-amino-cyclopropane-1-carboxylate synthase 2
AOC2	Allene oxide cyclase 2
AOS	Allene oxide synthase
ATL	Arabidopsis Tóxicos en Levadura
BAT5	Bile acid transporter 5
BTL10	BCA2Â zinc finger ATL 10
bZIP	Basic leucine zipper
CAC	Clathrin adaptor complex
CaMBP	Calmodulin-binding protein
CBP	Calcium binding protein
CBP60g	CaMBP 60-like G
CCD	Canadian Clubroot Differential
CR	Clubroot resistant
CS	Clubroot susceptible
Ct	Cycle threshold
CYP79F1	Cytochrome p450 79f1
CYP83A1	Cytochrome P450, family 83, subfamily A, polypeptide 1
CYP94B1	Cytochrome P450, family 94, subfamily B, polypeptide 1
CYP94C1	Cytochrome P450, family 94, subfamily C, polypeptide 1
dai	Days after inoculation
DAMP	Damage-associated molecular pattern
DEG	Differentially expressed gene
dH2O	Distilled water
DI	Disease index
DIR6	Dirigent protein 6
E2	E2 ubiquitin-conjugating enzyme
E3	E3 ubiquitin ligase
EREBP	APETALA2/ethylene-responsive element binding protein
ERF	Ethylene response factor
ET	Ethylene
ETI	Effector-triggered immunity
FAD7	Fatty acid desaturase 7
GDI1	Dissociation inhibitor 1
ICS	Isochorismate synthase
JA	Jasmonic acid
JA-Ile	Jasmonoyl-L-isoleucine
log_2_FC	Log_2_ fold-change
MAPK	Mitogen-activated protein kinase
MEKK	MAPK kinase kinase
MKK	MAPK kinase
MPK6	Mitogen-activated protein kinase 6
MYB15	MYB domain protein 15
NLP	Necrosis and ethylene-inducing peptide 1-like protein
NPR1	Nonexpresser of PR genes 1
NPR3	NPR1-like protein 3
OPR1	12-oxophytodienoate reductase 1
PAMP	Pathogen-associated molecular pattern
PCA	Principal component analysis
PEPR2	Pep 1 receptor 2
PLSP2A	Plastidic type I signal peptidase 2A
PR	Pathogenesis-related
PR1	Pathogenesis-related gene 1
PRR	Pattern recognition receptor
PTI	PAMP-triggered immunity
qRT-PCR	Quantitative Real-Time PCR
QTL	Quantitative resistance loci
R gene	Resistance gene
RBOH	Respiratory burst oxidase homolog
RIN	RNA integrity number
RLP	Receptor-like protein
RNA-seq	RNA sequencing
ROS	Reactive oxygen species
RPKM	Reads per kb of transcript per million mapped reads
SA	Salicylic acid
SARD1	SAR deficient 1
SCFE1	Sclerotinia culture filtrate elicitor 1
SOBIR1	Protein suppressor of BIR1–1
SRA	Sequence Read Archive
TF	Transcription factor
TGA	TGACG motif-binding protein
TIR-NBS-LRR	Toll-interleukin receptor nucleotide-binding site-leucine-rich repeat
TUA5	Tubulin alpha-5
UBC	Ubiquitin conjugating enzyme
VHA-E1	Vacuolar ATP synthase subunit E1
WAVH1	WAV3 homolog 1
