# Supplementary figures and images for: Comparative Transcriptome Analysis of Rutabaga (Brassica napus) Cultivars Indicates Activation of Salicylic Acid and Ethylene-Mediated Defenses in Response to Plasmodiophora brassicae
Source: Int J Mol Sci. 2020 Nov 8;21(21):8381. doi: 10.3390/ijms21218381 (PMC7664628; doi:10.3390/ijms21218381)

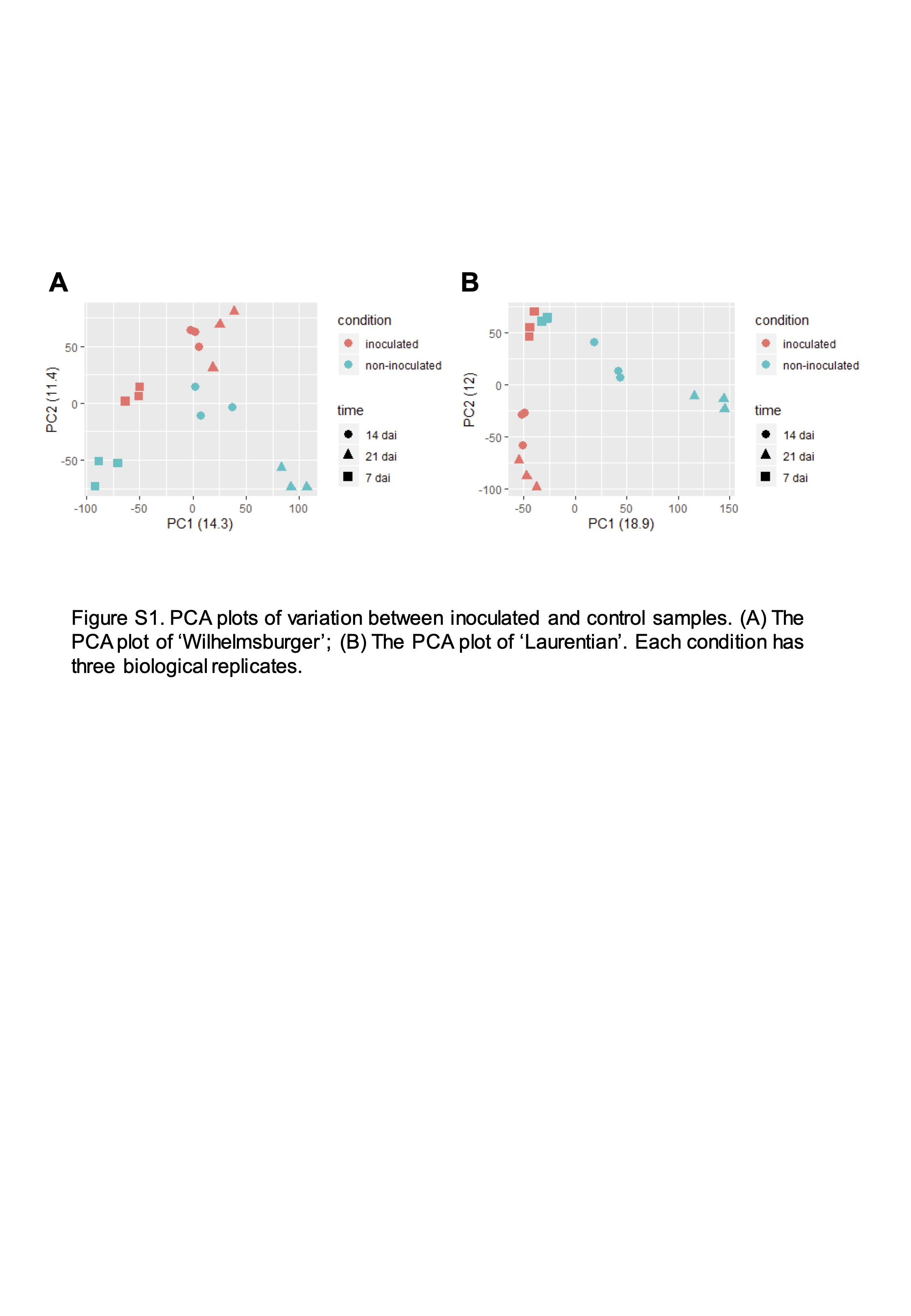

Supplement: Supplementary file 1 [file ijms-21-08381-s001.zip › Supplementary files/Figure S1.tiff]

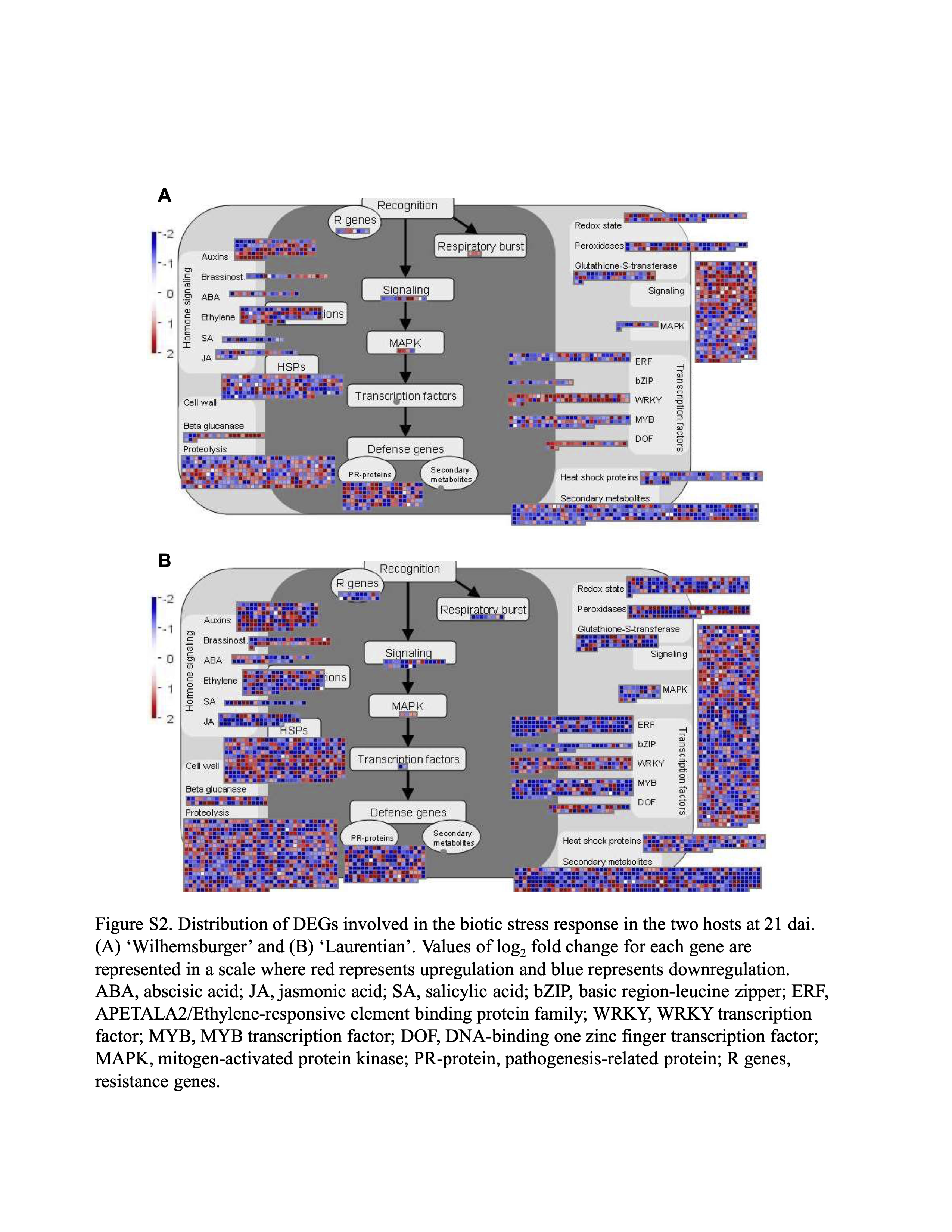

Supplement: Supplementary file 1 [file ijms-21-08381-s001.zip › Supplementary files/Figure S2.tiff]
